# Relationship between tumor biomarkers and efficacy in MARIANNE, a phase III study of trastuzumab emtansine ± pertuzumab versus trastuzumab plus taxane in HER2-positive advanced breast cancer

**DOI:** 10.1186/s12885-019-5687-0

**Published:** 2019-05-30

**Authors:** Edith A. Perez, Sanne Lysbet de Haas, Wolfgang Eiermann, Carlos H. Barrios, Masakazu Toi, Young-Hyuck Im, Pier Franco Conte, Miguel Martin, Tadeusz Pienkowski, Xavier B. Pivot, Howard A. Burris, Sven Stanzel, Monika Patre, Paul Anthony Ellis

**Affiliations:** 1 Mayo Clinic, 4500 San Pablo Rd. S, Jacksonville, FL 32224 USA; 2 F. Hoffmann-La Roche Ltd, Grenzacherstrasse 124, 4070 Basel, Switzerland; 3 Interdisciplinary Oncology Center, Nussbaumstrasse 12, 80336 Munich, Germany; 4 PUCRS School of Medicine, Av. Ipiranga 6681, Porto Alegre, RS 90619-900 Brazil; 5 Graduate School of Medicine, Kyoto University, Yoshida-Konoe-cho, Sakyo-ku, Kyoto 606-8501 Japan; 6 Samsung Medical Centre, 81 Irwon-Ro Gangnam-gu, Seoul, 06351 South Korea; 7 Department of Surgery, Oncology and Gastroenterology, University of Padova and Istituto Oncologico Veneto, Via Gattamelata 64, 35128 Padova, Italy; 8 Instituto de Investigacion Sanitaria Gregorio Marañón, CIBERONC, GEICAM, Universidad Complutense, Avda. de Séneca, 2, 28040 Madrid, Spain; 9 Postgraduate Medical Education Center, ul. Marymoncka 99, 02-813 Warsaw, Poland; 10 Paul Strauss Cancer Center, 3 Rue de la Porte de l’Hôpital, BP 30042–67065 Strasbourg, France; 11 Sarah Cannon Research Institute and Tennessee Oncology, PLLC, 250 25th Ave N, Nashville, TN 37203 USA; 12 Guys Hospital and Sarah Cannon Research Institute, Great Maze Pond, London, SE1 9RT UK

**Keywords:** Biomarker, Metastatic breast cancer, HER2, MARIANNE, mRNA, *PIK3CA*, Progression-free survival, PTEN, Trastuzumab emtansine, T-DM1

## Abstract

**Background:**

The phase III EMILIA and TH3RESA trials demonstrated clinical benefits of trastuzumab emtansine (T-DM1) therapy in patients with previously treated HER2-positive metastatic breast cancer (MBC). Data from these and other trials showed that T-DM1–associated survival benefits were observed across biomarker subgroups tested in these trials. Prespecified, exploratory analyses of the phase III MARIANNE study examined the effects of HER2-related biomarkers on PFS in patients administered T-DM1 in the first-line MBC setting.

**Methods:**

In MARIANNE, patients with previously untreated HER2-positive MBC were randomized (1:1:1) to trastuzumab plus taxane, T-DM1 plus placebo, or T-DM1 plus pertuzumab. Biomarker subgroups included HER2 and HER3 mRNA expression levels (≤median vs. >median), HER2 staining intensity (IHC 3+ vs. 2+ vs. 0/1+), *PIK3CA* status (mutated vs. non-mutated), PTEN H-score (≤median vs. >median), and PTEN protein expression level (0 vs. 1+ vs. 2+ vs. 3+ vs. 4+). PFS was analyzed descriptively for each subgroup using Kaplan–Meier methodology. Additional exploratory post-hoc analyses evaluated the effects of HER2 heterogeneity. Multivariate analyses were also performed.

**Results:**

Median PFS was numerically longer for patients with HER2 mRNA levels >median versus ≤median across treatment arms. In general, there were no predictive biomarkers of benefit for either T-DM1 treatment arm; most hazard ratios were close to 1 with wide confidence intervals that included the value 1. Focal HER2 expression (IHC 3+ or IHC 2+) was present in 3.8% of patients and was associated with numerically shorter PFS in the T-DM1–containing treatment arms versus trastuzumab plus taxane. Compared with non-mutated *PIK3CA*, mutated *PIK3CA* was associated with numerically shorter median PFS across treatment groups. Post-hoc multivariate analysis showed HER2 mRNA expression and mutated *PIK3CA* were prognostic for PFS (*P* ≤ 0.001 for both biomarkers).

**Conclusions:**

In MARIANNE, biomarkers related to the HER2 pathway did not have predictive value for PFS when comparing T-DM1 (with or without pertuzumab) with trastuzumab plus taxane. However, HER2 mRNA level and *PIK3CA* mutation status showed prognostic value. Evaluation of other potential biomarkers, including immune markers, is ongoing.

**Trial registration:**

Registration number: NCT01120184. Date of registration: April 28, 2010 (registered prospectively).

**Electronic supplementary material:**

The online version of this article (10.1186/s12885-019-5687-0) contains supplementary material, which is available to authorized users.

## Background

Therapeutic agents directed against human epidermal growth factor receptor 2 (HER2) have been developed to target the 15–20% of breast tumors that overexpress the HER2 protein [1–3]. Trastuzumab emtansine (T-DM1) is an antibody–drug conjugate that links the anti-HER2 humanized monoclonal antibody trastuzumab to DM1, a cytotoxic microtubule inhibitor. The trastuzumab portion delivers DM1 directly to HER2-overexpressing tumor cells, minimizing off-target effects [4]. Like trastuzumab, T-DM1 inhibits HER2 shedding, induces antibody-dependent cellular cytotoxicity, and inhibits phosphoinositide 3-kinase (PI3K)/Akt pathway-mediated cell signaling [5].

T-DM1 is indicated in many countries for single-agent treatment of patients with HER2-positive metastatic breast cancer (MBC) previously treated with trastuzumab and a taxane (separately or in combination). The clinical benefits of single-agent T-DM1 therapy in patients with previously treated advanced breast cancer were established in the phase III EMILIA and TH3RESA trials [6, 7]. In EMILIA, T-DM1 was compared with capecitabine plus lapatinib in patients with HER2-positive MBC previously treated with trastuzumab and a taxane (separately or in combination). In TH3RESA, T-DM1 was compared with treatment of physician’s choice (TPC) in patients previously treated with trastuzumab and lapatinib in the advanced setting and a taxane in any setting. In both studies, T-DM1 was associated with statistically significant improvements in both progression-free survival (PFS) and overall survival (OS) relative to the respective control regimen [6–8].

Given the survival benefits conferred by T-DM1 in previously treated MBC [6–8], the phase III MARIANNE study sought to explore the efficacy and safety of T-DM1 in patients with previously untreated HER2-positive advanced breast cancer [9]. Patients in MARIANNE were randomized 1:1:1 to trastuzumab plus taxane (hereafter referred to as “control”), T-DM1 plus placebo (hereafter referred to as “T-DM1”), or T-DM1 plus pertuzumab (T-DM1 + P). Median PFS was 13.7 months in the control group compared with 14.1 months and 15.2 months in the T-DM1 and T-DM1 + P groups, respectively. Based on these results, T-DM1 treatment (with or without pertuzumab) resulted in non-inferior, but not superior, PFS relative to control (stratified hazard ratio [HR] = 0.91, 97.5% confidence interval [CI] 0.73–1.13, *P* = 0.31 for T-DM1 vs. control; 0.87, 97.5% CI 0.69–1.08, *P* = 0.14 for T-DM1 + P vs. control).

The relationship between the expression of specific biomarkers and efficacy of T-DM1 has been examined in EMILIA [10], TH3RESA [11], and a series of phase II trials investigating patients with HER2-positive MBC [12–14]. In general, the collective data from these studies demonstrated that there were no predictive biomarkers for T-DM1 survival benefits. These studies also suggested that high expression of HER2 mRNA levels (greater than median levels [>median]) was a positive prognostic marker, associated with both a better PFS and OS benefit in EMILIA. However, although a predictive value of HER2 mRNA for T-DM1 treatment was observed in both EMILIA and THERESA, results were not fully consistent regarding endpoints observed, and may have been influenced by factors like treatment line and comparator (control) regimen. The relevance of HER2 mRNA prompted questions about whether HER2 expression heterogeneity impacts treatment response.

Although PI3K pathway aberrations have been implicated in resistance to HER2-targeted therapies such as trastuzumab [15, 16] and lapatinib [17], results from biomarker analyses of EMILIA study data have shown that T-DM1 was associated with numerical improvements in median PFS, median OS, and overall response rate relative to capecitabine plus lapatinib, irrespective of *PI3K catalytic subunit alpha* (*PIK3CA*) mutation status [10]. Preclinical studies (cell lines and xenograft models) have also shown that T-DM1 exhibits potent activity in the presence of *PIK3CA* mutations [10]. Conversely, among patients randomized to capecitabine plus lapatinib in EMILIA, median PFS and OS were numerically shorter in patients with *PIK3CA*-mutated versus *PIK3CA*–non-mutated tumors [10]. In TH3RESA, T-DM1–treated patients had similar median PFS regardless of *PIK3CA* mutation status. Median PFS was also comparable in TPC-treated patients with tumors expressing mutated versus non-mutated *PIK3CA*, of which 80% received trastuzumab-based treatment [11].

In MARIANNE, the impact of hormone receptor status on PFS has been reported previously, and no differences in PFS were seen between patients who were hormone receptor–positive and –negative [9]. However, the MARIANNE study also provides an opportunity to examine whether T-DM1 treatment of previously untreated HER2-positive MBC could overcome the poor prognosis of mutated *PIK3CA*, assess the prognostic and predictive value of HER2 mRNA, and potentially identify associations with other biomarkers. To this end, the purpose of the exploratory analyses presented herein was to examine the association between biomarkers related to the HER2 pathway and PFS in patients from the phase III MARIANNE study.

## Methods

### Study design and patients

The MARIANNE (NCT01120184) study design and primary analysis results have been published elsewhere [9]. In brief, MARIANNE is a phase III, international study of patients with centrally-confirmed HER2-positive unresectable, progressive, or recurrent locally advanced or previously untreated MBC (advanced breast cancer) and no prior treatment for advanced disease. Patients were randomized (1:1:1) to control (trastuzumab plus taxane [docetaxel or paclitaxel]), T-DM1, or T-DM1 + P.

The primary efficacy endpoint was PFS, which was determined by an independent review committee (IRC). PFS was defined as the time from randomization to the first occurrence of IRC-assessed disease progression (per Response Evaluation Criteria in Solid Tumors version 1.1 [18]) or death from any cause. Protocol-specified exploratory biomarker analyses were conducted to evaluate the association between PFS and molecular markers related to its mechanism of action (e.g., HER pathway effectors).

The MARIANNE study protocol, including a description of the exploratory biomarker analyses, was approved by the institutional review board/ethics committee at each participating center. The trial conformed to Good Clinical Practice guidelines, the Declaration of Helsinki, and applicable local laws. All patients provided written informed consent, which included consent for biomarker analyses. Analysis of PTEN protein expression required additional tissue and therefore a separate written patient consent. These analyses were conducted only in patients who provided this consent.

### Biomarker assessments

Samples from the primary or metastatic tumor were submitted to a central laboratory for the determination of HER2 status by immunohistochemistry (IHC; PATHWAY anti-HER-2/neu [4B5] assay, Ventana Medical Systems, Inc.) and in situ hybridization (INFORM HER2 Dual ISH assay, Ventana Medical Systems, Inc.). HER2 distribution was also assessed (not prespecified); patients with either IHC 2+ or IHC 3+ staining were categorized according to whether HER2 staining was focal (10–29%), heterogeneous (30–79%), or homogeneous (≥80%), based on the total percentage of cells stained with 2+ and 3+ intensity. Tissue samples used for HER2 testing were also used for additional biomarker analyses.

HER2 mRNA level, HER3 mRNA level, HER2 staining intensity, *PIK3CA* status, PTEN H-score, and PTEN protein level were all prespecified as biomarkers for inclusion in this exploratory analysis. Analysis of PTEN protein expression required a separate written patient consent, as described above, and optional donation of additional tumor samples, which were provided as additional material from the same tissue sample originally provided.

The methods used for the biomarker assessments have been described in detail elsewhere [10, 11]. Briefly, HER2 and HER3 mRNA expression levels were measured using quantitative real-time polymerase chain reaction (cobas® 4800 System, Roche Molecular Diagnostics) and reported as a ratio in reference to glucose-6-phosphate dehydrogenase expression. *PIK3CA* mutation status was determined using the cobas® *PIK3CA* Mutation Test (Roche Molecular Diagnostics) and cobas® 4800 System (Roche Molecular Diagnostics). The analysis of cytoplasmic PTEN protein expression was assessed via IHC (138G6 rabbit monoclonal antibody, Cell Signaling Technology®). The analysis of *PIK3CA* mutation status was performed at HistoGeneX NV (Berchem, Belgium). Central HER2 testing was performed by multiple pathologists at Targos Molecular Pathology GmbH (Kassel, Germany). Additional biomarker analyses were also performed by Targos Molecular Pathology GmbH.

### Statistical methods

This exploratory analysis evaluated the potential prognostic and predictive value of HER2 mRNA expression level (≤median vs. >median), HER3 mRNA expression level (≤median vs. >median), HER2 staining intensity (IHC 3+ vs. 2+ vs. 0/1+), *PIK3CA* status (mutated vs. non-mutated), PTEN H-score (≤median vs. >median), and PTEN protein level (0 vs. 1+ vs. 2+ vs. 3+ vs. 4+) as biomarkers for PFS. Predictive biomarker effects were evaluated based on PFS HRs and associated CIs within biomarker-defined subgroups, while prognostic effects were evaluated across treatment arms. PFS was analyzed descriptively for each biomarker subgroup using the Kaplan–Meier method. A Cox proportional hazards regression model was used to estimate HRs and 97.5% CIs (choice of CI coverage probability is due to the hierarchical statistical testing procedure employed in this study, applying parallel statistical testing of T-DM1 vs. control and T-DM1 + P vs. control, see [9]).

Biomarker status was evaluated based on baseline tumor samples. As in prior T-DM1 studies [10–14, 19], biomarker subgroups for HER2 and HER3 were defined using the median distribution values for mRNA expression (≤median vs. >median). To further understand the association between HER2 expression and PFS, patients were also grouped by the intensity of HER2 staining (IHC 3+ vs. 2+ vs. 0/1+). For the analysis of *PIK3CA*, tumors were classified as either mutated (presence of ≥1 mutation) or non-mutated. PTEN subgroups were defined using the median H-score [20] value (≤median vs. >median) and predefined protein expression categories assigned based on the percentage of tumor cells with cytoplasmic staining relative to adjacent, internal, non-tumor control cells (0 [no staining] vs. 1+ [decreased] vs. 2+ [slightly decreased] vs. 3+ [equal] vs. 4+ [increased]). A post-hoc analysis was conducted to understand whether HER2 expression heterogeneity affected PFS. In this analysis, patients with IHC 2+ and IHC 3+ staining were grouped by HER2 distribution (focal, heterogeneous, or homogeneous). In addition, a data-driven (post-hoc) analysis of PFS was performed in which the subgroup of patients with at least one biomarker shown to negatively impact treatment response to T-DM1 were compared with the subgroup comprised of the remaining study patients. Additionally, the impact of the combination of both HER2 mRNA (≤median and > median) and *PIK3CA* mutation (mutated and non-mutated) on PFS was evaluated in combination subgroups as part of the post-hoc data-driven analyses.

The effect of HER2 mRNA (analyzed as a categorical variable using the median mRNA expression value as a cut-off) and *PIK3CA* status (mutated vs. non-mutated) on PFS was also examined in a post-hoc multivariate analysis using two Cox proportional hazards regression models. The first model, which was fully adjusted, included the following additional covariates: study treatment, prior treatment with taxane and/or trastuzumab (yes/no), prior treatment with anthracyclines (yes/no), world region, presence/absence of visceral disease, presence/absence of measurable disease, disease-free interval, baseline Eastern Cooperative Oncology Group performance status score, hormone receptor status, therapy setting, breast cancer staging, and menopausal status. The second multivariate analysis approach also started with the fully adjusted model, then a stepwise backward variable selection procedure was applied. A *P*-value threshold of 0.05 was used to decide whether a covariate should be retained in the model or removed. The treatment effect was kept “fixed” (i.e., study treatment was not allowed to be removed from the model at any step). Goodness of fit of the two models was assessed by computing Akaike’s Information Criterion (AIC) [21]. Smaller AIC values indicate relatively better model performance. As all data reported herein derive from exploratory analyses, the *P*-values as well as the HRs and associated 95% CIs from the multivariate analyses are provided only for illustrative purposes and only for the model with the smaller AIC value.

## Results

### Patients and demographics

A total of 365 patients were randomized to control, 367 to T-DM1, and 363 to T-DM1 + P. HER2 status was determined using samples derived from the primary tumor for 77% (844/1095) of patients and from a metastatic tumor for 11% (123/1095) of patients; tumor location (primary vs. metastatic) was not known in 12% (128/1095) of patients. Biomarker availability was generally balanced across treatment arms (Table 1), with ≥90.0% of patients providing data for the analyses of HER2 mRNA, HER3 mRNA, and *PIK3CA* status. Because the evaluation of PTEN protein expression was performed on an additional tissue sample, the donation of which was optional and required separate written consent, samples from fewer (49.5%) patients were available for the analysis of this biomarker. Distribution of tumor location (primary, metastatic, or unknown) was similar for the assessment of each biomarker (HER2 mRNA, HER3 mRNA, *PIK3CA* status, and PTEN) and was well balanced across the three treatment arms (data not shown). Median baseline values for HER2 mRNA, HER3 mRNA, and PTEN protein expression and the proportion of patients with tumors expressing mutated *PIK3CA* (26–28%) were equally distributed across treatment arms. HER2 distribution was equally distributed across the treatment arms; for example, 3%, 3%, and 4% of patients in the control, T-DM1, and T-DM1 + P arms, respectively, had focal HER2 distribution (Table 1).

**Table 1:** Biomarker status at baseline among patients in the intent-to-treat population of the MARIANNE study

	Trastuzumab + taxane (Control) (*N* = 365)^a^	T-DM1 (T-DM1) (*N* = 367)^a^	T-DM1 + pertuzumab (T-DM1 + P) (*N* = 363)^a^
HER2 mRNA			
n (%)	330 (90)	339 (92)	330 (91)
Median (range)	57.28 (1.9–4182.1)	56.89 (0.9–3615.6)	61.61 (2.1–3281.2)
HER2 status, n (%)			
ISH-positive/IHC 3+	294 (81)	305 (83)	295 (81)
ISH-positive/IHC 2+	27 (7)	25 (7)	27 (7)
ISH-positive/IHC 1+, 0 or unknown	5 (1)	2 (1)	3 (1)
ISH-negative/IHC 3+	7 (2)	5 (1)	7 (2)
ISH-negative or unknown/IHC 2+	0	0	2 (1)
ISH unknown/IHC 3+	32 (9)	30 (8)	29 (8)
HER3 mRNA			
n (%)	331 (91)	342 (93)	332 (91)
Median (range)	1.88 (0.1–33.1)	1.57 (0.1–52.7)	1.71 (0.1–26.0)
*PIK3CA* status			
n (%)	326 (89)	332 (90)	328 (90)
Mutated, n (%)	83 (25)	92 (28)	88 (27)
Non-mutated, n (%)	243 (75)	240 (72)	240 (73)
PTEN H-score			
n (%)	182 (50)	181 (49)	179 (49)
Median (range)	135.0 (0.0–310.0)	180.0 (0.0–370.0)	160.0 (0.0–380.0)
HER2 distribution			
n (%)^b^	360 (99)	365 (99)	360 (99)
Focal (10–29%), n (%)	14 (4)	12 (3)	15 (4)
Heterogeneous (30–79%), n (%)	35 (10)	37 (10)	33 (9)
Homogeneous (≥80%), n (%)	311 (86)	316 (87)	312 (87)

^a^Intention-to-treat population

^b^Only includes patients who were IHC 2+ or IHC 3 +

Biomarker prevalence reported here is based on the number of patients with data available for each marker. *HER2* human epidermal growth factor receptor 2, *IHC* immunohistochemistry, *ISH* in situ hybridization, *PIK3CA* phosphoinositide 3-kinase catalytic subunit alpha, *P* pertuzumab, *PTEN* phosphatase and tensin homolog, *T-DM1* trastuzumab emtansine

### Impact of biomarker expression on PFS

**Fig. 1:**
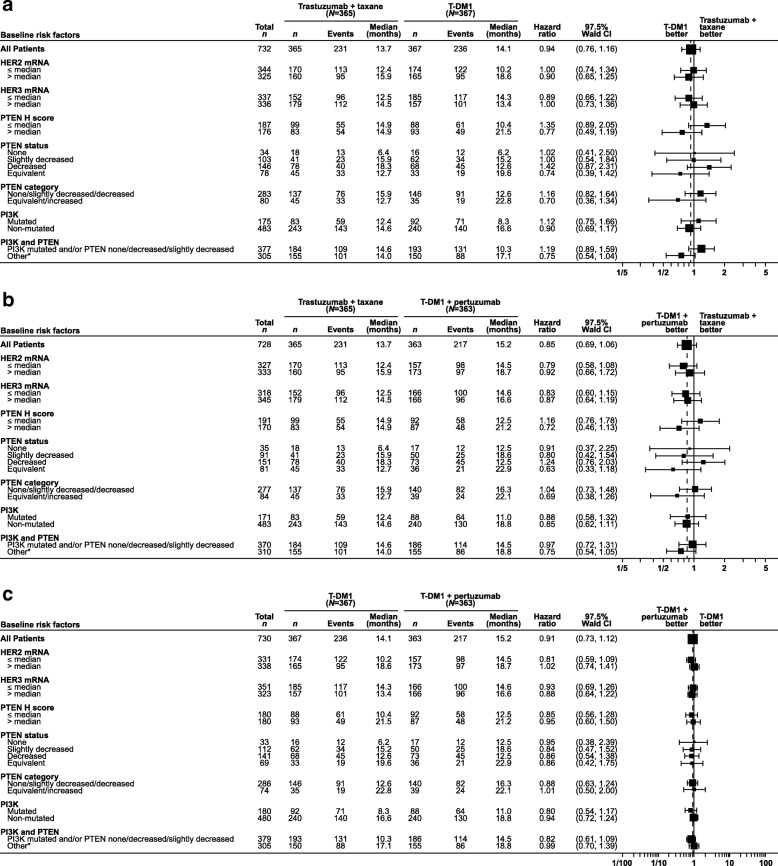
PFS by biomarker status. **a** T-DM1 versus control. **b** T-DM1 plus pertuzumab versus control. **c** T-DM1 plus pertuzumab versus T-DM1. *CI* confidence interval, *HER* human epidermal growth factor receptor, *HR* unstratified hazard ratio, *PFS* progression-free survival, *PI3K* phosphoinositide 3-kinase, *PTEN* phosphatase and tensin homolog, *T-DM1* trastuzumab emtansine. *PI3K and PTEN category “other” represents patients with no PI3K mutation and no decrease in PTEN, or one of these in combination with an unknown for the other marker (e.g., no PI2K mutation and unknown PTEN status)

Of the biomarkers examined, in general, none revealed a subgroup with clear treatment benefit for T-DM1 or T-DM1 + P relative to control with regard to PFS (Fig. 1a and b). HRs comparing PFS between T-DM1 and T-DM1 + P versus control within each biomarker subgroup remained close to the overall HRs for these treatment comparisons, except for PTEN H-score > median. No biomarker subgroup was associated with substantially longer median PFS for T-DM1 + P versus T-DM1, with all HRs clustered around the overall HR of 0.81 (Fig. 1c). Review of these data should consider that biomarker subgroup sample sizes were small for some subgroups and the CIs for the respective HRs were wide and included the value 1.

**Fig. 2:**
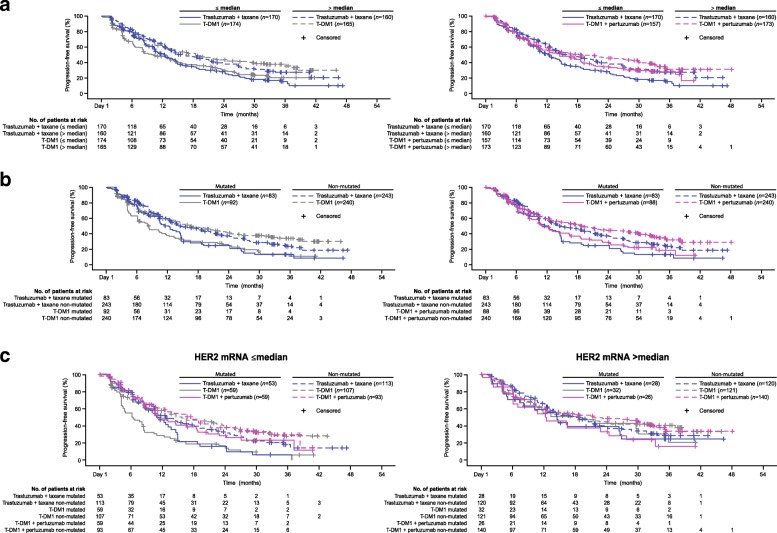
Statistical analysis of PFS in subgroups defined by **a** HER2 mRNA expression (≤median vs. >median), **b** *PIK3CA* status (mutated vs. non-mutated), and **c** *PIK3CA* status in combination with HER2 mRNA expression. See Additional file 1: Tables S2 and S3 for a table of PFS values and hazard ratios. *CI* confidence interval, *HER* human epidermal growth factor receptor, *HR* unstratified hazard ratio, *PIK3CA* phosphoinositide 3-kinase catalytic subunit alpha, *PFS* progression-free survival, *T-DM1* trastuzumab emtansine

In all treatment arms, HER2 mRNA levels >median versus ≤median were associated with numerically longer PFS (control: 15.9 vs. 12.4 months; T-DM1: 18.6 vs. 10.2 months; T-DM1 + P: 18.7 vs. 14.5 months) (Fig. 2a). Thus, HER2 mRNA was found to be prognostic of PFS benefit within all treatment arms. Expression of mutated versus non-mutated *PIK3CA* was associated with numerically shorter PFS within all three treatment arms (Fig. 2b).

Despite these differences, as noted above, HER2 mRNA and *PIK3CA* did not have predictive value for a treatment benefit in response to T-DM1 compared with control, as none of the biomarker subgroups in the population of patients treated with T-DM1 had a greater PFS benefit compared with the control arm; however, some numerical differences between treatment arms were notable. Among patients with HER2 mRNA ≤median, median PFS was numerically higher for T-DM1 + P versus control, but not for T-DM1 versus control. Among patients with >median HER2 mRNA, median PFS was numerically higher for both T-DM1 treatment arms compared with control. In the analysis of *PIK3CA*, median PFS was numerically higher in the control arm compared with both T-DM1 treatment arms for patients with mutated *PIK3CA*. By contast median PFS was numerically lower in the control arm compared with the T-DM1 treatment arms for those with non-mutated *PIK3CA*.

Patients who received T-DM1 (alone or in combination with pertuzumab) had a median PFS that was numerically larger, with PTEN H-scores >median (21.5 and 21.2 months, respectively) versus those with H-scores ≤median (10.4 and 12.5 months, respectively) (Fig. 1). For patients who received control, there was no numerical difference in median PFS by PTEN protein level status (14.9 months in patients with PTEN H-score > median and ≤ median values). The PTEN categories (e.g., none/slightly decreased/decreased vs. equivalent/increased) did not show a consistent trend in association with PFS outcome across treatment arms.

### Impact of HER2 expression levels and heterogeneity on PFS

Median PFS was numerically longer in patients with IHC 3+ versus IHC 2+ HER2 staining; this difference was more pronounced among patients randomized to T-DM1 or T-DM1 + P versus control (Table 2).

**Table 2:** Progression-free survival by HER2 expression subgroups

	Trastuzumab + taxane (Control)		T-DM1			T-DM1 + pertuzumab			
			(T-DM1)			(T-DM1 + P)			
	No. patients / No. patients with PFS event	Median PFS (mo)	No. patients / No. patients with PFS event	Median PFS (mo)	HR vs. trastuzumab + taxane (97.5% CI)^a^	No. patients / No. patients with PFS event	Median PFS (mo)	HR vs. trastuzumab + taxane (97.5% CI)^a^	HR vs. T-DM1 + placebo (97.5% CI)^a^
All patients^b^									
IHC 3+	333/209	14.4	340/215	14.6	0.93	331/195	16.7	0.83	0.90
					(0.75–1.16)			(0.67–1.04)	(0.72–1.12)
IHC 2+	27/19	12.6	25/20	7.3	1.13	29/20	8.3	1.25	0.98
					(0.55–2.32)			(0.61–2.59)	(0.48–2.02)
IHC 2+/3+ patients combined^c^									
Focal IHC 2+/3+ (10–29%)^d^	14/8	12.4	12/10	6.4	1.51	15/12	7.5	1.41	1.00
					(0.52–4.40)			(0.50–3.94)	(0.38–2.65)
Heterogeneous IHC 2+/3+ (30–79%)	35/27	10.6	37/25	8.3	1.04	33/20	6.3	1.11	0.91
								(0.57–2.17)	(0.46–1.78)
					(0.55–1.94)				
Homogeneous IHC 2+/3+ (≥80%)	311/193	14.6	316/200	14.7	0.92	312/183	17.8	0.82	0.89
								(0.65–1.04)	(0.71–1.13)
					(0.74–1.16)				
IHC 3+ patients only									
Focal IHC 3+ (10–29%)^d^	9/5	8.3	11/7	8.3	1.20	8/7	4.2	5.11	2.28
					(0.32–4.50)			(0.99–26.40)	(0.60–8.71)
Heterogeneous IHC 3+ (30–79%)	44/29	10.5	45/34	10.0	1.15	29/16	17.8	0.79	0.65
					(0.65–2.03)			(0.39–1.60)	(0.33–1.29)
Homogeneous IHC 3+ (≥80%)	280/175	14.6	284/174	15.2	0.89	294/172	17.7	0.82	0.92
					(0.70–1.14)			(0.65–1.05)	(0.73–1.17)

^a^Unstratified hazard ratio^b^ Five patients with IHC 0/1+ and five patients with unknown IHC status are not included in this table

^c^Categories were based on IHC subgroup and then combined

^d^Compared with the overall population, samples with focal HER2 expression were more likely to express mutated *PIK3CA* and lower levels of HER2 mRNA

*CI* confidence interval, *HER2* human epidermal growth factor receptor 2, *HR* hazard ratio, *IHC* immunohistochemistry, *NE* not estimable, *P* pertuzumab, *PFS* progression-free survival, *PIK3CA* phosphoinositide 3-kinase catalytic subunit alpha, *T-DM1* trastuzumab emtansine

The association between HER2 expression heterogeneity and PFS was examined in a post-hoc, data-driven, exploratory analysis. Of the 1004 patients with IHC 3+ HER2 staining, 28 (2.8%) exhibited focal expression, 118 (11.8%) exhibited heterogeneous expression, and 858 (85.5%) exhibited homogeneous expression. When 2+ and 3+ staining intensity was considered in IHC2+ or IHC3+ patients, homogeneous staining was associated with numerically longer median PFS than focal/heterogeneous staining. For IHC 3+ patients, for whom only 3+ intensity staining was considered, focal HER2 expression was present in a small subgroup of patients (3.8% [41/1085]), and was associated with numerically shorter median PFS in the two T-DM1 treatment arms versus the control arm (Table 2).

### Post-hoc, data-driven analysis of subgroups defined by the presence/absence of biomarkers with negative impact on PFS

**Fig. 3:**
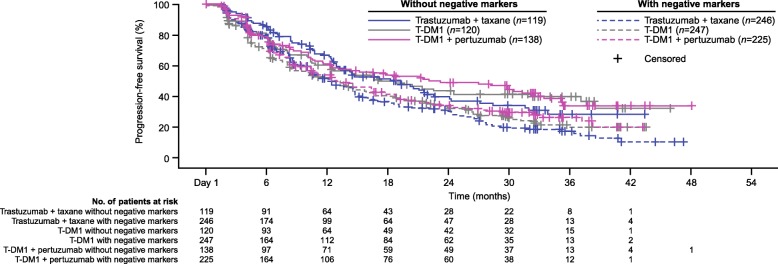
Kaplan–Meier curve of PFS in subgroups defined by the presence/absence of negatively impacting biomarkers;*.*Biomarkers were considered negatively prognostic of response to HER2-targeted treatment based on their association with a numerical decrease in PFS, specifically, these included expression of mutated *PIK3CA*, low HER2 mRNA level (≤median), and focal HER2 distribution. Patients without negative markers were those with non-mutated *PIK3CA*, high HER2 mRNA levels (>median), and non-focal (i.e., heterogeneous or homogenous) distribution of HER2. Patients with negative markers were those with mutated *PIK3CA*, low HER2 mRNA levels (≤median), and focal HER2 distribution. *HER2* human epidermal growth factor receptor 2, *PIK3CA* phosphoinositide 3-kinase catalytic subunit alpha, *PFS* progression-free survival, *T-DM1* trastuzumab emtansine

Given the numerical decrease in PFS associated with *PIK3CA* mutation, low HER2 mRNA expression (≤median), and focal HER2 expression, especially in patients receiving trastuzumab emtansine, a post-hoc, data-driven analysis of PFS was performed evaluating patient subgroups based on the combined presence and absence of these biomarkers (Fig. 3). In this analysis, the Kaplan–Meier curves of all three treatment arms were largely overlapping for those patients with selected biomarkers, and for those without. For all three treatment groups, however, patients without these selected biomarkers (i.e., those with non-mutated *PIK3CA*, >median HER2 mRNA expression, and non-focal HER2 expression) experienced better PFS independent of treatment arm. Therefore, this analysis did not reveal a subset of patients who experienced treatment benefit in terms of PFS when receiving T-DM1.

### Post-hoc analysis of HER2 mRNA and PIK3CA mutation subgroups in patients with de novo MBC

In MARIANNE, 38% of patients (410/1088) had de novo disease (defined as patients diagnosed with metastatic disease within 30 days from the date of initial diagnosis), with the percentage of patients with de novo MBC being balanced between treatment arms (36.4% control, 39.8% T-DM1, and 36.1% T-DM1 + P). The tumor tissue collected from these patients was fresh (i.e., not archival) and had not been affected by prior treatments; therefore, it should reflect a reliable biomarker status for the analysis of treatment effects (see Additional file 1: Figure S1). Interpretation of these results showed that the prognostic value of HER2 mRNA and *PIK3CA* was inconsistent between the two T-DM1 treatment arms. Therefore, these results were inconclusive. However, it should be acknowledged that the biology of de novo disease may differ from that of previously treated disease.

### Multivariate analysis evaluating the effects of HER2 mRNA expression and *PIK3CA* status on PFS

Compared with the fully adjusted multivariate model, the stepwise backward variable selection model was better in terms of goodness of model fit (i.e., had a smaller AIC value). Per the final model resulting from applying the stepwise backward variable selection procedure, HER2 mRNA expression and *PIK3CA* mutation status were both prognostic for PFS (*P* ≤ 0.001 for both). The *P*-value for the treatment effect was *P* = 0.057. The complete list of covariates that remained in the final model and associated *P*-values as well as the HRs and associated 95% CIs is summarized in Additional file 1: Table S1.

### Overlap between *PIK3CA* status, HER2 mRNA expression, and HER2 IHC staining pattern and impact on PFS

Given the contradicting results on the prognostic value of *PIK3CA* mutation in MARIANNE versus TH3RESA and EMILIA, further evaluation of *PIK3CA* mutation status and overlap with other biomarkers was undertaken. It should be noted that these analyses and interpretation of these results are limited due to being post-hoc and data-driven and because they are investigating effects in a sub-subgroup (i.e., a smaller and more selected group of patients). Of the patients with both HER2 IHC expression and *PI3KCA* mutation status available, a higher *PIK3CA* mutation rate was observed in patients with focal or heterogeneous HER2 expression (41.9%; 57/136) compared to those with homogeneous HER2 expression (24.0%; 202/840) (Table 3). Among 501 patients with ≤median HER2 mRNA levels, 484 also had *PIK3CA* mutation status available; in 35.3% (171/484) of these patients, *PIK3CA* was mutated. This was higher than the prevalence in the 467 of 498 patients who had >median HER2 mRNA levels and *PIK3CA* mutation status available; of these 18.4% (86/467) had mutated *PIK3CA* (Table 3). The efficacy of single-agent T-DM1 seemed to be independent of *PIK3CA* status in the subgroup of patients with HER2 mRNA >median (Fig. 2c) in contrast to patients with HER2 mRNA ≤median. However, for T-DM1 + P, the impact of *PIK3CA* mutation seemed less different between the HER2 mRNA subgroups than that observed with single-agent T-DM1.

**Table 3:** Overlap of HER2 mRNA level, *PI3K* mutation status, and HER2 IHC staining pattern biomarker subgroups

Biomarker subgroup			Patients n (%^a^)
HER2 mRNA and *PI3K* status available (*N* = 951)	HER2 mRNA level	*PI3K* mutation status	
	≤median (*n* = 484)	Mutated	171 (35.3)
		Non-mutated	313 (64.7)
	>median (*n* = 467)	Mutated	86 (18.4)
		Non-mutated	381 (81.6)
HER2 mRNA and HER2 IHC expression available (*N* = 989)	HER2 mRNA level	HER2 IHC expression	
	≤median (*n* = 495)	Focal/heterogeneous	114 (23.0)
		Homogeneous	381 (77.0)
	>median (*n* = 494)	Focal/heterogeneous	19 (3.8)
		Homogeneous	475 (86.6)
*PI3K* mutation status and HER2 IHC expression available (*N* = 976)	HER2 IHC expression	*PI3K* mutation status	
	Focal/heterogeneous (*n* = 136)	Mutated	57 (41.9)
		Non-mutated	79 (58.1)
	Homogeneous (*n* = 840)	Mutated	202 (24.0)
		Non-mutated	638 (76.0)

^a^Percentage based on number of patients with both biomarkers available and calculated within subgroups. HER2 mRNA missing *n* = 96; PI3K mutation status missing *n* = 109; IHC H score missing n = 10

*HER2* human epidermal growth factor receptor 2, *IHC* immunohistochemistry, *PI3K* phosphoinositide 3-kinase

## Discussion

In this exploratory analysis of biomarker data from the phase III MARIANNE study, none of the evaluated biomarkers (all of which are related to the HER2 pathway) showed predictive value for PFS benefit with T-DM1 treatment alone or in combination with pertuzumab versus control in this group of patients with HER2-positive advanced breast cancer previously untreated in the advanced setting. HER2 mRNA expression was prognostic of PFS, as HER2 mRNA expression levels >median were associated with numerically longer median PFS across all treatment arms. *PIK3CA* status was also prognostic, with mutated *PIK3CA* associated with numerically shorter median PFS for all study regimens compared with non-mutated *PIK3CA*. The prognostic value of HER2 mRNA expression and *PIK3CA* status was confirmed in a post-hoc multivariate analysis.

The finding that HER2 mRNA levels >median were prognostic for clinical benefit of HER2-targeted therapy is consistent with the biomarker analysis from the CLEOPATRA study in which patients received first-line treatment of HER2-positive MBC with pertuzumab plus trastuzumab plus docetaxel or placebo plus trastuzumab plus docetaxel (control) [22]. In CLEOPATRA, high HER2 mRNA expression was associated with better clinical outcome based on data pooled across treatment arms. Interestingly, in the exploratory biomarker analyses of EMILIA and TH3RESA, which recruited patients with previously treated HER2-positive MBC, single-agent T-DM1 was also associated with numerically longer PFS in the subgroup of patients with higher versus lower levels of HER2 mRNA [10, 11]. However, in the control arm of TH3RESA (TPC), but not EMILIA (capecitabine plus lapatinib), median PFS was similar for patients with ≤median and > median HER2 mRNA expression. Therefore, the ability to differentiate clinical benefit derived from T-DM1 versus control in the high HER2 mRNA subgroup seemed to depend on treatment line (first line vs. later line) and comparator regimen.

Across treatment arms, we did not observe a consistent trend for the PTEN. Among T-DM1 treated patients, there was a trend toward numerically greater PFS with higher PTEN H-scores, however, this was not observed in the control group. Multiple studies have evaluated the association between PTEN protein expression and response to anti-HER2 therapy and many also have shown no influence of PTEN expression on outcome. In both TH3RESA and EMILIA, patients with higher and lower PTEN protein levels experienced a PFS benefit from T-DM1 [10, 11]. The phase III North Central Cancer Treatment Group (NCCTG) N9831 trial compared doxorubicin and cyclophosphamide followed by weekly paclitaxel (arm A) with the same regimen plus a year of sequential trastuzumab (arm B) or concurrent trastuzumab (arm C, with trastuzumab started on the same day as weekly paclitaxel). This study found a benefit of adjuvant trastuzumab on disease-free survival for patients with HER2-positive breast cancer, independent of tumor PTEN status [23]. In the CLEOPATRA study, there was a consistent PFS benefit from pertuzumab plus trastuzumab plus docetaxel versus placebo plus trastuzumab plus docetaxel regardless of PTEN status; however, low cytoplasmic PTEN was associated with a worse OS outcome for the pertuzumab arm [22].

In this biomarker analysis of MARIANNE, as well as the biomarker analysis of CLEOPATRA [22], mutated *PIK3CA* was associated with worse clinical outcome in terms of PFS across all treatment arms, and the difference in PFS between patients with mutated and non-mutated *PIK3CA* was greatest in the T-DM1 treatment arms. In contrast, in both EMILIA and TH3RESA, the positive effects of T-DM1 on clinical outcome were observed in patients with mutated and non-mutated *PIK3CA* [10, 11]. The reasons for the discrepant results across these phase III studies are unclear. However, patients in MARIANNE did not receive prior treatment for advanced disease, while patients in EMILIA and TH3RESA were pretreated. This may have influenced the biomarker status, which was assessed from archival samples from the primary tumor in 80% of patients in the EMILIA and TH3RESA trials and, as a result, may not reflect the biomarker status at study start in a later-line disease setting. In MARIANNE, more patients had non-archival tissue, as 37% of patients had de novo disease. Another explanation for the conflicting observations could be the association between mutant *PIK3CA* and HER2 mRNA expression levels in the tumors of MARIANNE patients, which was observed in a post-hoc analysis evaluating overlap of biomarkers across patients. In MARIANNE, it was observed that a *PIK3CA* mutation was much more prevalent in tumors with low HER2 mRNA expression (35.3%) compared with those with high HER2 mRNA expression (18.4%). In line with these associations, a higher *PIK3CA* mutation rate was seen in focal or heterogeneous (41.9%) versus homogeneous (24.0%) tumors. A similar association was observed in CLEOPATRA (unpublished data), suggesting that this phenotype may be genuine and does not reflect a bias in biomarker prevalence among MARIANNE patients. Higher prevalence of *PIK3CA* mutation in low HER2 mRNA expressing tumors was not seen in EMILIA and TH3RESA, but it is possible that analyses resulted in patient subgroups with imbalances in biomarker expression, as *PIK3CA* mutation data were not available for all patients in those trials.

**Fig. 4:**
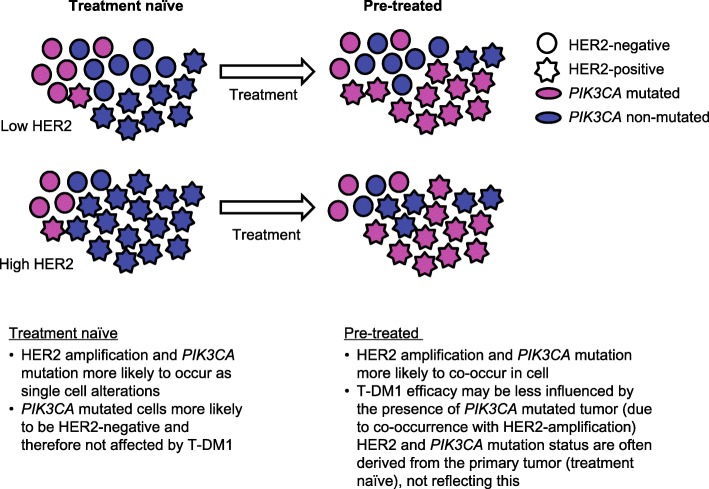
Hypothesized influence of treatment history on co-occurrence of mutated *PIK3CA* and HER2 overexpression. *HER* human epidermal growth factor receptor, *PIK3CA* phosphoinositide 3-kinase catalytic subunit alpha, *T-DM1* trastuzumab emtansine

Although this finding from MARIANNE should be interpreted with caution, one could hypothesize a plausible biological rationale for this discrepancy. It is unknown whether the co-occurrence of these poor prognostic markers is found only at the tumor level, or if it is also found at the single-cell level. Findings from an in situ single-cell analysis suggest that, while mutated *PIK3CA* and HER2 gene amplifications occurs individually in HER2-positive breast cancer, following chemotherapy, the cell population containing both alterations was enriched in patients with resistant disease [24]. One could hypothesize that treatment history influences the co-occurrence of mutated *PIK3CA* and HER2 overexpression on a single-cell level (Fig. 4). In a treatment-naive tumor, HER2 might be heterogeneously amplified, with *PIK3CA* mutation occurring more commonly in HER2-negative rather than HER2-positive cells, which makes a tumor with low HER2 expression (as observed in tumors with greater HER2 heterogeneity) more likely to also show a *PIK3CA* mutation. However, following treatment, *PIK3CA* mutation may be enriched in HER2-amplified tumor cells (e.g., as a resistance mechanism to HER2-targeted therapy). Thus, a *PIK3CA* mutation would be detected in the primary tumor, but the impact of *PIK3CA* mutation on clinical benefit when receiving T-DM1 may be different in these two situations. As T-DM1 binds to HER2, theoretically, T-DM1 is more likely to kill a *PIK3CA*-mutated tumor cell with co-occurring HER2 amplification versus a HER2-negative, *PIK3CA*-mutated cell, despite the fact that the tumor as a whole is HER2-positive and *PIK3CA*-mutated. Our post-hoc analyses assessing T-DM1 treatment effects in patients with *PIK3CA* mutated tumors and a high versus low HER2 mRNA level seem to support this hypothesis, but no firm conclusions can be drawn from these exploratory analyses. As hypothesized above, the genetic composition of the tumor may change from the first-line to later-line setting and affect the impact of the *PIK3CA* mutation on outcome in different disease settings, as observed between the MARIANNE, EMILIA and TH3RESA studies.

Because patients randomized to T-DM1–based treatment in MARIANNE did not receive systemic chemotherapy, HER2 heterogeneity was hypothesized to be a main driver of response in T-DM1–treated patients. Evaluation of the HER2 staining patterns in MARIANNE showed that focal or heterogeneous HER2 staining patterns were infrequently seen in patients with IHC 3+ tumors (2.8 and 11.8%, respectively), whereas a much higher proportion of patients with IHC 2+ tumors (48.1%) exhibited focal staining. These findings are consistent with previous studies, in which more HER2 heterogeneity was reported in IHC 2+ versus IHC 3+ tumors [25–27]. In a data-driven, post-hoc analysis, we categorized patients according to the pattern of HER2 staining using the total of cells stained with 2+ and 3+ intensity, and found median PFS to be numerically shorter in T-DM1–treated patients with focal versus homogeneous HER2 expression (6.4 vs. 14.7 months for T-DM1 and 7.5 vs. 17.8 months for T-DM1 + P). We observed low levels of HER2 gene amplification in patients with heterogeneous HER2 staining in MARIANNE, a phenomenon that has previously been reported [26]. Moreover, it is known that intra-tumoral heterogeneity commonly results from chromosomal instability, which is itself associated with poor prognosis [28]. Thus, the numerically shorter median PFS observed in MARIANNE patients with tumors exhibiting non-homogeneous HER2 distribution may relate to both insufficient target (owing to low HER2 gene copy number/expression) and to the fact that heterogeneous tumors are inherently unstable (and thus predisposed to poorer clinical outcomes). The shorter median PFS in focal subgroups was more pronounced in patients who received T-DM1–containing treatment compared with the control arm, which may suggest a larger impact of an insufficient target in the absence of systemic chemotherapy.

As noted above, the evaluation of co-occurrence of biomarkers in MARIANNE showed that focal/heterogeneous tumors were more likely to be *PIK3CA*-mutated (41.9%) versus those with homogeneous HER2 expression (24.0%) and that tumors with low (≤median) HER2 mRNA levels were also more likely to be *PIK3CA*-mutated (35.3%) compared with tumors with high (>median) HER2 mRNA levels (18.4%). These observations suggest that there may be multiple cancer drivers within individual tumors and that these can, in turn, influence treatment response. While this phenomenon is intriguing, the expression of multiple, negative prognostic biomarkers within a tumor makes it difficult to isolate the individual contribution of each biomarker to clinical outcomes. To understand if a more beneficial outcome could be achieved in T-DM1–containing treatment arms when biomarker subgroups with a hypothesized negative impact on clinical outcome were excluded from the evaluation, we conducted a post-hoc, data-driven analysis in which the PFS of patients with tumors characterized by the presence of non-mutated *PIK3CA*, high HER2 mRNA levels, and heterogeneous or homogeneous distribution of HER2 was compared to the PFS of all other patients. In this analysis, median PFS was comparable across treatment arms in patients with the positive biomarkers, suggesting that even if patients expressing negative prognostic biomarkers had been initially excluded from MARIANNE, PFS outcomes in T-DM1–treated patients would probably still not have been superior to those observed in patients treated with control. Additional studies are needed to further evaluate the impact of negatively prognostic biomarkers on the treatment effects of HER2-targeted therapy as well as to understand the co-occurrence of such biomarkers on a single-cell level and their associations with clinical outcome across treatment lines. Such an analysis would require serial sampling (i.e., samples taken at primary diagnosis, recurrence, start of first-line treatment, etc.) and development of more sensitive assays that allow the analysis of tumor markers in blood, which is an easier way to collect serial samples.

This exploratory analysis may be limited by the source of the tumor samples; approximately 80% of the tissue used in the MARIANNE biomarker analyses sampled from primary tumors, with only ~ 10% deriving from metastatic tumors. Biomarker status may have changed between the primary and metastatic stages. However, the use of primary tumor data is common, and biomarker analyses of archival samples have shown that, for example, HER2 mRNA expression is associated with clinical outcome in T-DM1–treated patients across different lines of therapy [10, 14, 22, 29], suggesting that HER2 is relatively stable throughout the course of disease. This may however differ for other markers. Another limitation was the small sample sizes for some analyses–particularly the data-driven, post-hoc exploratory analyses examining the co-occurrence of biomarkers–preclude definitive conclusions; thus, the data presented herein should be considered hypothesis-generating. In addition, our analyses only examined the impact of HER2-related biomarkers on the primary efficacy endpoint of MARIANNE (PFS), but not on OS, as OS data were immature at the time of the MARIANNE primary analysis. Last, evaluation of other potentially relevant biomarkers, such as studies measuring immune markers, has yet to be performed.

## Conclusions

In the phase III MARIANNE study, biomarkers related to the HER2 pathway (HER2 and HER3 mRNA expression levels, *PIK3CA* mutation status, PTEN H-score and protein expression level, and tumor heterogeneity) were not predictive of PFS benefit in MBC patients administered first-line treatment with T-DM1 with or without pertuzumab versus trastuzumab plus a taxane. However, both HER2 mRNA level and *PIK3CA* mutation status were shown to have prognostic value across all treatment arms in both univariate and multivariate analyses. Several hypotheses on biomarkers relating to the HER2 pathway, their overlap, evolution over time, and impact on treatment outcome have been generated. Further research, including the identification of patient characteristics that may distinguish HER2-targeted treatment responders from non-responders and analysis of the influence of tumor-infiltrating lymphocytes and immune markers, is ongoing.

## Additional files

Additional file 1: **Table S1.** List of covariates (and associated hazard ratios with 95% CIs as well as *P*-values) included in the final multivariate regression model applied to evaluate PFS. Final model derived using stepwise backward variable selection. **Table S2.** PFS in HER2 mRNA and *PIK3CA* biomarker subgroups. **Table S3.** PFS in biomarker subgroups defined by *PIK3CA* in combination with HER2 mRNA expression. **Figure S1.** PFS by HER2 mRNA expression and PIK3CA biomarker status in patients with de novo disease. (PDF 546 kb)

Additional file 2: List of institutional review boards/ethics committees. (PDF 354 kb)

## Abbreviations

AIC: Akaike’s Information Criterion
CI: Confidence interval
HER2: Human epidermal growth factor receptor 2
HR: Hazard ratio
IHC: Immunohistochemistry
IRC: Independent review committee
ISH: In situ hybridization
MBC: Metastatic breast cancer
NE: Not estimable
OS: Overall survival
PFS: Progression-free survival
PI3K: Phosphoinositide 3-kinase
*PIK3CA*: *PI3K catalytic subunit alpha*
PTEN: Phosphatase and tensin homolog
T-DM1: Trastuzumab emtansine
TPC: Treatment of physician’s choice
