# Correction to: Relationship between tumor biomarkers and efficacy in MARIANNE, a phase III study of trastuzumab emtansine ± pertuzumab versus trastuzumab plus taxane in HER2-positive advanced breast cancer

**DOI:** 10.1186/s12885-019-5831-x

**Published:** 2019-06-24

**Authors:** Edith A. Perez, Sanne Lysbet de Haas, Wolfgang Eiermann, Carlos H. Barrios, Masakazu Toi, Young-Hyuck Im, Pier Franco Conte, Miguel Martin, Tadeusz Pienkowski, Xavier B. Pivot, Howard A. Burris, Sven Stanzel, Monika Patre, Paul Anthony Ellis

**Affiliations:** 1 Mayo Clinic, 4500 San Pablo Rd. S, Jacksonville, FL 32224 USA; 2 F. Hoffmann-La Roche Ltd, Grenzacherstrasse 124, 4070 Basel, Switzerland; 3 Interdisciplinary Oncology Center, Nussbaumstrasse 12, 80336 Munich, Germany; 4 PUCRS School of Medicine, Av. Ipiranga 6681, Porto Alegre, RS 90619-900 Brazil; 5 Graduate School of Medicine, Kyoto University, Yoshida-Konoe-cho, Sakyo-ku, Kyoto, 606-8501 Japan; 6 Samsung Medical Centre, 81 Irwon-Ro Gangnam-gu, Seoul, 06351 South Korea; 7 Department of Surgery, Oncology and Gastroenterology, University of Padova and Istituto Oncologico Veneto, Via Gattamelata 64, 35128 Padova, Italy; 8 Instituto de Investigacion Sanitaria Gregorio Marañón, CIBERONC, GEICAM, Universidad Complutense, Avda. de Séneca, 2, 28040 Madrid, Spain; 9 Postgraduate Medical Education Center, ul. Marymoncka 99, 02-813 Warsaw, Poland; 10 Paul Strauss Cancer Center, 3 Rue de la Porte de l’Hôpital, BP 30042, 67065 Strasbourg, France; 11 Sarah Cannon Research Institute and Tennessee Oncology, PLLC, 250 25th Ave N, Nashville, TN 37203 USA; 12 Guys Hospital and Sarah Cannon Research Institute, Great Maze Pond, London, SE1 9RT UK

**Correction to: BMC Cancer**

**https://doi.org/10.1186/s12885-019-5687-0**

Following publication of the original article [1], the authors reported the following errors in the article.

1. In Table 2, the layout has been updated. The corrected Table 2 is supplied below:
2. The legend for Fig. 3 has been adapted for clearer readability. The updated legend is as follows:
3. The competing interests statement has been updated below.

**Table 2:** Progression-free survival by HER2 expression subgroups

	Trastuzumab + taxane (Control)		T-DM1 (T-DM1)			T-DM1 + pertuzumab (T-DM1+P)			
	No. patients / No. patients with PFS event	Median PFS (mo)	No. patients / No. patients with PFS event	Median PFS (mo)	HR vs. trastuzumab + taxane (97.5% CI)^a^	No. patients / No. patients with PFS event	Median PFS (mo)	HR vs. trastuzumab + taxane (97.5% CI)^a^	HR vs. T-DM1 + placebo (97.5% CI)^a^
All patients^b^									
IHC 3+	333/209	14.4	340/215	14.6	0.93 (0.75–1.16)	331/195	16.7	0.83 (0.67–1.04)	0.90 (0.72–1.12)
IHC 2+	27/19	12.6	25/20	7.3	1.13 (0.55–2.32)	29/20	8.3	1.25 (0.61–2.59)	0.98 (0.48–2.02)
IHC 2+/3+ patients combined^c^									
Focal IHC 2+/3+ (10–29%)^d^	14/8	12.4	12/10	6.4	1.51 (0.52–4.40)	15/12	7.5	1.41 (0.50–3.94)	1.00 (0.38–2.65)
Heterogeneous IHC 2+/3+ (30–79%)	35/27	10.6	37/25	8.3	1.04 (0.55–1.94)	33/20	6.3	1.11 (0.57–2.17)	0.91 (0.46–1.78)
Homogeneous IHC 2+/3+ (≥80%)	311/193	14.6	316/200	14.7	0.92 (0.74–1.16)	312/183	17.8	0.82 (0.65–1.04)	0.89 (0.71–1.13)
IHC 3+ patients only									
Focal IHC 3+ (10–29%)^d^	9/5	8.3	11/7	8.3	1.20 (0.32–4.50)	8/7	4.2	5.11 (0.99–26.40)	2.28 (0.60–8.71)
Heterogeneous IHC 3+ (30–79%)	44/29	10.5	45/34	10.0	1.15 (0.65–2.03)	29/16	17.8	0.79 (0.39–1.60)	0.65 (0.33–1.29)
Homogeneous IHC 3+ (≥80%)	280/175	14.6	284/174	15.2	0.89 (0.70–1.14)	294/172	17.7	0.82 (0.65–1.05)	0.92 (0.73–1.17)

^a^Unstratified hazard ratio

^b^Five patients with IHC 0/1+ and five patients with unknown IHC status are not included in this table

^c^Categories were based on IHC subgroup and then combined

^d^Compared with the overall population, samples with focal HER2 expression were more likely to express mutated PIK3CA and lower levels of HER2 mRNA *CI* confidence interval, *HER2* human epidermal growth factor receptor 2, *HR* hazard ratio, *IHC* immunohistochemistry, *NE* not estimable, *P* pertuzumab, *PFS* progression-free survival, *PIK3CA* phosphoinositide 3-kinase catalytic subunit alpha, *T-DM1* trastuzumab emtansine

**Fig. 3:**
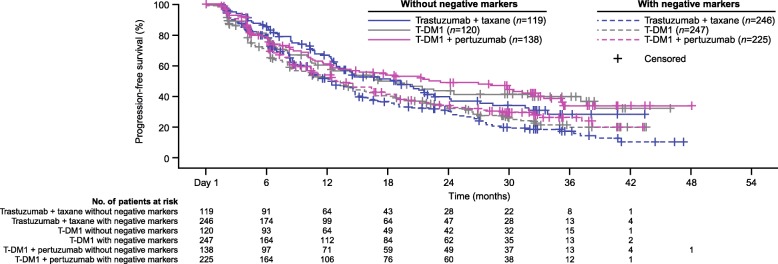
Kaplan–Meier curve of PFS in subgroups defined by the presence/absence of negatively impacting biomarkers.* *Biomarkers were considered negatively prognostic of response to HER2-targeted treatment based on their association with a numerical decrease in PFS. Specifically, these included expression of mutated PIK3CA, low HER2 mRNA level (≤median), and focal HER2 distribution. Patients without negative markers were those with nonmutated PIK3CA, high HER2 mRNA levels (>median), and non-focal (i.e., heterogeneous or homogenous) distribution of HER2. Patients with negative markers were those with mutated PIK3CA, low HER2 mRNA levels (≤median), and focal HER2 distribution. *HER2* human epidermal growth factor receptor 2, *PIK3CA* phosphoinositide 3-kinase catalytic subunit alpha, *PFS* progression-free survival, *T-DM1* trastuzumab emtansine
