# Supplementary material for: Relationship between tumor biomarkers and efficacy in MARIANNE, a phase III study of trastuzumab emtansine ± pertuzumab versus trastuzumab plus taxane in HER2-positive advanced breast cancer
Source: BMC Cancer. 2019 May 30;19:517. doi: 10.1186/s12885-019-5687-0 (PMC6543580; doi:10.1186/s12885-019-5687-0)
Supplement: Supplementary file 1 — Table S1. List of covariates (and associated hazard ratios with 95% CIs as well as P-values) included in the final multivariate regression model applied to evaluate PFS. Final model derived using stepwise backward variable selection. Table S2. PFS in HER2 mRNA and PIK3CA biomarker subgroups. Table S3. PFS in biomarker subgroups defined by PIK3CA in combination with HER2 mRNA expression. Figure S1. PFS by HER2 mRNA expression and PIK3CA biomarker status in patients with de novo disease. (PDF 546 kb) [file 12885_2019_5687_MOESM1_ESM.pdf]

ADDITIONAL FILE 1

Supplement to: Perez EA, de Haas SL, Eiermann W, et al. Relationship between tumor biomarkers and efficacy in MARIANNE, a phase III study of trastuzumab emtansine ± pertuzumab versus trastuzumab plus taxane in HER2-positive advanced breast cancer.

Table S1 List of covariates (and associated hazard ratios with 95% CIS as well as P-values) included in the final multivariate regression model applied to evaluate PFS. Final model derived using stepwise backward variable selection

Covariate[*]	Hazard ratio (95% CI)	*P* value
HER2 mRNA expression (reference: >median)[†]		
≤median	1.38 (1.17, 1.63)	<0.0001
PIK3CA mutation status (reference: non-mutated)[†]		
Mutated	1.56 (1.31, 1.86)	<0.0001
Treatment comparison (reference: trastuzumab + taxane)[‡]		0.0569
T-DM1	0.87 (0.72, 1.05)	0.1449
T-DM1+P	0.80 (0.66, 0.96)	0.0175
Presence visceral disease (reference: no)		
Yes	1.31 (1.09, 1.58)	0.0043
Baseline ECOG performance status score (reference: 0)		0.0013
1	1.35 (1.15, 1.58)	0.0003
2[§]	0.98 (0.14, 7.07)	0.9867
Presence of measurable disease at baseline (reference: no)		
Yes	1.90 (1.50, 2.41)	<0.0001
Therapy setting (reference: metastatic)		
Locally advanced breast cancer	0.55 (0.32, 0.95)	0.0334
Prior treatment with taxane and/or trastuzumab (reference: no)		
Yes	1.27 (1.04, 1.55)	0.0187
World region (reference: Western Europe, Canada, Australia/Pacific)		0.0307
Asia	1.07 (0.86, 1.32)	0.5445

Eastern Europe	1.22 (0.97, 1.53)	0.0968
USA	1.09 (0.84, 1.41)	0.5256
Others	1.46 (1.15, 1.86)	0.0018
Disease-free interval (reference: ≤2 years)		0.0404
>2 years	0.77 (0.62, 0.95)	0.0150
De novo	0.89 (0.69, 1.15)	0.3665

[*]All covariates were evaluated as categorical variables.

[†]Data for missing values are not shown: 96 patients had unknown HER2 mRNA expression levels and 109 patients had unknown *PIK3CA* mutation status.

[‡]Study treatment was kept fixed in the model.

[§]Only two patients had an ECOG performance status score of 2.

CI confidence interval, *ECOG* Eastern Cooperative Oncology Group, *HER2* human epidermal growth factor receptor 2, *PIK3CA* phosphoinositide 3-kinase catalytic subunit alpha

Table S2 PFS in HER2 mRNA and *PIK3CA* biomarker subgroups

**HER2 mRNA expression (≤median vs. >median)**								
	**Trastuzumab + taxane** (control)		**T-DM1** (T-DM1)			**T-DM1 + pertuzumab** (T-DM1+P)		
	n	Median PFS (months)	n	Median PFS (months)	HR vs. trastuzumab + taxane (97.5% CI)	n	Median PFS (months)	HR vs. trastuzumab + taxane (97.5% CI)
HER2 mRNA ≤median	170	12.4	174	10.2	1.00 (0.74–1.34)	157	14.5	0.79 (0.58–1.08)
HER2 mRNA >median	160	15.9	165	18.6	0.90 (0.65–1.25)	173	18.7	0.92 (0.66–1.27)
***PIK3CA* status (mutated vs. non-mutated)**								
	**Trastuzumab + taxane** (control)		**T-DM1** (T-DM1)			**T-DM1 + pertuzumab** (T-DM1+P)		
	n	Median PFS (months)	n	Median PFS (months)	HR vs. trastuzumab + taxane (97.5% CI)	n	Median PFS (months)	HR vs. trastuzumab + taxane (97.5% CI)
Mutated *PIK3CA*	83	12.4	92	8.3	1.12 (0.75–1.66)	88	11.0	0.88 (0.58–1.32)
Non-mutated *PIK3CA*	243	14.6	240	16.6	0.90 (0.69–1.17)	240	18.8	0.85 (0.65–1.11)

CI confidence interval, *HER* human epidermal growth factor receptor, *HR* unstratified hazard ratio, *P* pertuzumab, *PIK3CA* phosphoinositide 3-kinase catalytic subunit alpha, *PFS* progression-free survival, *T-DM1* trastuzumab emtansine

Table S3 PFS in biomarker subgroups defined by *PIK3CA* in combination with HER2 mRNA expression

	**Trastuzumab + taxane** (control)		**T-DM1** (T-DM1)			**T-DM1 + pertuzumab** (T-DM1+P)			
	n	Median PFS (months)	n	Median PFS (months)	HR vs. trastuzumab + taxane (97.5% CI)	n	Median PFS (months)	HR vs. trastuzumab + taxane (97.5% CI)	HR vs. T-DM1 (97.5% CI)
Low HER2 mRNA									
Mutated *PIK3CA*	53	10.8	59	6.4	1.31 (0.81–2.13)	59	11.1	0.71 (0.43–1.19)	0.57 (0.35–0.91)
Non-mutated *PIK3CA*	113	12.6	107	15.8	0.84 (0.57–1.23)	93	17.5	0.80 (0.53–1.20)	0.96 (0.64–1.45)
High HER2 mRNA									
Mutated *PIK3CA*	28	14.6	32	18.6	0.88 (0.43–1.83)	26	12.4	1.13 (0.55–2.32)	1.31 (0.64–2.69)
Non-mutated *PIK3CA*	120	18.3	121	18.8	0.92 (0.63–1.36)	140	22.1	0.91 (0.62–1.33)	0.99 (0.68–1.45)

CI confidence interval, *HER* human epidermal growth factor receptor, *HR* unstratified hazard ratio, *P* pertuzumab, *PIK3CA* phosphoinositide 3-kinase catalytic subunit alpha, *PFS* progression-free survival, *T-DM1* trastuzumab emtansine

Fig. S1 PFS by HER2 mRNA expression and *PIK3CA* biomarker status in patients with de novo disease. (a) T-DM1 versus control. (b) T-DM1 plus pertuzumab versus control. (c) T-DM1 plus pertuzumab versus T-DM1. Median time to event estimated from Kaplan–Meier curves. Unstratified cox proportional hazards regression was used to estimate HRs and 97.5% CIs for each treatment arm comparison and covariate subgroup. Vertical dashed line shows the HR for all patients. The size of the squares is proportional to the sample size in the subgroup

a

Baseline risk factors	Total *n*	Trastuzumab + taxane (*N*=133) *n*	Events	Median (months)	T-DM1 (*N*=146) *n*	Events	Median (months)	Hazard ratio	97.5% Wald CI
**All Patients**	279	133	83	15.5	146	101	12.2	1.16	(0.83, 1.61)
**HER2 mRNA**									
≤ median	135	61	42	12.6	74	53	10.2	0.96	(0.60, 1.53)
> median	114	55	29	20.5	59	38	12.4	1.49	(0.86, 2.60)
**HER3 mRNA**									
≤ median	114	51	33	14.6	63	37	15.2	0.80	(0.47, 1.38)
> median	136	66	39	17.2	70	53	8.4	1.57	(0.98, 2.52)
**PI3K**									
Mutated	59	23	17	8.5	36	29	7.6	1.03	(0.52, 2.05)
Non-mutated	190	96	55	19.3	94	59	13.4	1.14	(0.75, 1.74)

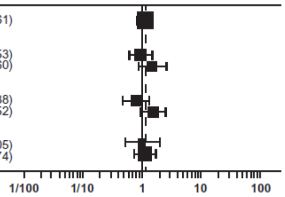

b

Baseline risk factors	Total *n*	Trastuzumab + taxane (*N*=133) *n*	Events	Median (months)	T-DM1 + pertuzumab (*N*=131) *n*	Events	Median (months)	Hazard ratio	97.5% Wald CI
**All Patients**	264	133	83	15.5	131	75	18.8	0.80	(0.56, 1.15)
**HER2 mRNA**									
≤ median	116	61	42	12.6	55	36	14.6	0.78	(0.47, 1.30)
> median	118	55	29	20.5	63	29	30.5	0.85	(0.47, 1.53)
**HER3 mRNA**									
≤ median	107	51	33	14.6	56	31	18.8	0.72	(0.41, 1.26)
> median	129	66	39	17.2	63	35	19.9	0.88	(0.52, 1.48)
**PI3K**									
Mutated	50	23	17	8.5	27	20	9.0	0.80	(0.38, 1.69)
Non-mutated	184	96	55	19.3	88	42	29.9	0.70	(0.44, 1.12)

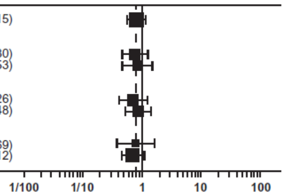

c

Baseline risk factors	Total *n*	T-DM1 (*N*=146) *n*	Events	Median (months)	T-DM1 + pertuzumab (*N*=131) *n*	Events	Median (months)	Hazard ratio	97.5% Wald CI
**All Patients**	277	146	101	12.2	131	75	18.8	0.71	(0.51, 1.00)
**HER2 mRNA**									
≤ median	129	74	53	10.2	55	36	14.6	0.83	(0.51, 1.35)
> median	122	59	38	12.4	63	29	30.5	0.58	(0.33, 1.01)
**HER3 mRNA**									
≤ median	119	63	37	15.2	56	31	18.8	0.86	(0.50, 1.49)
> median	133	70	53	8.4	63	35	19.9	0.58	(0.36, 0.95)
**PI3K**	63	36	29	7.6	27	20	9.0	0.79	(0.41, 1.51)
Mutated	182	94	59	13.4	88	42	29.9	0.64	(0.41, 1.02)
Non-mutated									

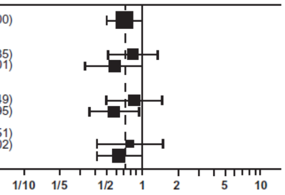

CI confidence interval, *HER2* human epidermal growth factor receptor 2, *HR* unstratified hazard ratio, *PI3K* phosphoinositide 3-kinase, *T-DM1* trastuzumab emtansine
