# Supplementary material for: Relationship between tumor biomarkers and efficacy in MARIANNE, a phase III study of trastuzumab emtansine ± pertuzumab versus trastuzumab plus taxane in HER2-positive advanced breast cancer
Source: BMC Cancer. 2019 May 30;19:517. doi: 10.1186/s12885-019-5687-0 (PMC6543580; doi:10.1186/s12885-019-5687-0)
Supplement: Supplementary file 2 — List of institutional review boards/ethics committees. (PDF 354 kb) [file 12885_2019_5687_MOESM2_ESM.pdf]

ADDITIONAL FILE 3

Supplement to: Perez EA, de Haas SL, Eiermann W, et al. Relationship between tumor biomarkers and efficacy in MARIANNE, a phase III study of trastuzumab emtansine ± pertuzumab versus trastuzumab plus taxane in HER2-positive advanced breast cancer.

List of Institutional Review Boards/Independent Ethics Committees:

- Comité de Docencia e investigación, Sanatorio de la Providencia, San Miguel de Tucuman, Argentina
- Comite Independiente De Etica Para Ensayos En Farmacolog, Buenos Aires, Argentina (2 sites)
- Hunter New England Human Research Ethics Committee, Research Support and Development Office, Hunter New England Area Health District, New Lambton, New South Wales, Australia
- Ethics Committee, Peter MacCallum Cancer Centre, East Melbourne, Victoria, Australia
- Bellberry Human Research Ethics Committee, Bellberry Limited, Dulwich, South Australia, Australia
- Royal Adelaide Hospital Research Ethics Committee, Royal Adelaide Hospital, Adelaide, Australia
- Ethikkomission der stadt Wien, Wien, Austria (2 sites)
- The Public Hospitals Authority, Ministry of Health, Nassau, Bahamas
- Commissie Medische Ethiek GZA, GasthuisZusters Antwerp (GZA), Wilrijk, Belgium (3 sites)
- Ethics Committee University Hospital Centre Sarajevo, University Hospital Centre Sarajevo, Sarajewo, Bosnia and Herzegovina
- Ethics Committee University Clinical Centre of the Republic of Srpska, University Clinical Centre of the Republic of Srpska, Banja Luka, Bosnia and Herzegovina
- Comitê De Ética Em Pesquisa Em Seres Humanos, Centro De Referência De Saúde Da Mulher, São Paulo, Brazil
- Comitê de Ética em Pesquisa da PUCRS, PUCRS, Porto Alegre, Brazil
- "Comitê de Ética em Pesquisa em Seres Humanos da Universidade do Vale do Itajaí - UNIVALI/SC, Universidade do Vale do Itajaí - UNIVALI/SC, Itajaí, Brazil
- CEP, Hospital Nossa Senhora da Conceição, Porto Alegre, Brazil
- Comissão de Ética para Análise de Projetos de Pesquisa, University of São Paulo Faculty of Medicine Clinics Hospital, São Paulo, Brazil

- Comitê de Ética em Pesquisa do Hospital Moinhos de Vento, Pesquisa do Hospital Moinhos de Vento, Porto Alegre, Brazil
- Comitê de Ética em Pesquisa – INCA, Instituto Nacional de Câncer (INCA), Rio de Janeiro, Brazil
- Health Research Ethics Board of Alberta (HREBA) – Cancer Committee, Alberta Innovates – Health Solutions, Edmonton, Alberta, Canada
- Comité d'éthique de la recherche du CHU de Québec, CHU de Québec, Quebec, Canada
- CHUS Hopital Fleurimont EC/IRB, CHUS Hopital Fleurimont, Sherbrooke, Quebec, Canada
- Ontario Cancer Research Ethics Board, MaRS Centre, Toronto, Ontario, Canada
- Comite De Etica De Investigacion, Instituto Cancerologico de Nariño, Pasto, Colombia
- Comite De Etica De Investigacion Clinica, Clinica del Country, Bogota, Colombia
- Eticka komise Masarykova onkologickeho ustavu, Masarykova onkologickeho ustavu, Brno, Czech Republic
- Eticka Komise Fakultni Nemocnice Olomouc, Fakultni Nemocnice Olomouc, Olomouc, Czech Republic
- Eticka komise Nemocnice Na Bulovce, Nemocnice Na Bulovce, Prague, Czech Republic
- Eticka komise pro multicentricka hodnoceni, Multicentricka hodnoceni, Prague, Czech Republic
- Den Videnskabsetiske komité for Region Syddanmark, Region Syddanmark, Syddanmark, Denmark
- Comité de protection des personnes (CPP) Est II, Besançon, France (12 sites)
- Ethikkommission München, Ludwig-Maximilians Universität, Munich, Germany (8 sites)
- National Ethics Committee, Athens, Greece (2 sites)
- Comité de Ética Independiente Zugueme, Guatemala City, Guatemala (2 sites)
- Medical Research Council, Ethics Committee for Clinical Pharmacology, Semmelweis University, Budapest, Hungary (4 sites)
- Comitato Etico Provinciale Modena, Azienda USL di Modena, Modena, Italy
- Comitato Etico Di Area Vasta Romagna E Irst, Istituto Scientifico Romagnolo per lo Studio e la Cura dei Tumori (I.R.S.T.) S.r.l., Meldola, Italy (2 sites)
- Comitato Etico Interaziendale della Provincia di Messina, Azienda Ospedaliera Universitaria, Messina, Italy
- Comitato Etico Provinciale Di Reggio Emilia, Azienda Unita Sanitaria Locale di Reggio Emilia, Reggio Emilia, Italy

- Ce Azienda Sanitaria Provinciale Di Catania, Azienda Sanitaria Provinciale Di Catania, Reggio Calabria, Italy
- Comitato Etico Regionale Unico CERU, EGAS Ente per la gestione accentrata dei servizi condivisi, Udine, Italy
- Comitato Etico, IRCCS Fondazione Maugeri Di Pavia, Pavia, Italy
- Comitato Etico, IRCCS Istituto Clinico Humanitas, Rozzano (Milan), Italy
- Ce Sperimentazione Clinica Dei Medicinali, Oncologia Medica, Ospedale Misericordia E Dolce; Prato, Italy
- Comitato Etico delle Aziende Sanitarie dell'Umbria, Aziende Sanitarie dell'Umbria, Perugia, Italy
- Comitato Etico Degli, IRCCS Istituto Europeo di Oncologia e Centro Cardiologico Monzino, Milan, Italy
- Comitato Etico Regione Calabria Sezione Area Centro, Magna Graecia University, Catanzaro, Italy
- Iwate Medical University School of Medicine, Morioka, Japan
- Tohoku University Hospital, Sendai, Japan
- Saitama Medical University International Medical Center, Hidaka, Japan
- Tokyo Medical University Hospital, Tokyo, Japan
- Tokai University Hospital, Isehara, Japan
- Osaka University Hospital, Osaka, Japan
- National Hospital Organization Osaka National Hospital, Osaka, Japan
- Hyogo Cancer Center, Akashi, Japan
- Sagara Hospital, Kagoshima, Japan
- Niigata Cancer Center Hospital, Niigata, Japan
- Saitama Cancer Center, Saitama, Japan
- National Cancer Center Hospital, Tokyo , Japan
- The Cancer Institute Hospital of JFCR, Tokyo, Japan
- Shizuoka Cancer Center, Shizuoka, Japan
- Kanazawa University Hospital, Kanazawa, Japan
- Gifu University Hospital, Gifu, Japan
- Aichi Cancer Center Hospital, Nagoya, Japan
- Kyoto University Hospital, Kyoto, Japan

- Hyogo College Of Medicine, Nishinomiya, Japan
- National Hospital Organization Shikoku, Matsuyama, Japan
- National Hospital Organization Kyushu Cancer Center, Fukuoka, Japan
- Kumamoto University Hospital, Kumamoto, Japan
- Kumamoto City Hospital, Kumamoto, Japan
- Kawasaki Medical School Hospital, Kurashiki, Japan
- Shizuoka General Hospital, Shizuoka, Japan
- Hiroshima University Hospital, Hiroshima, Japan
- Toranomon Hospital, Tokyo, Japan
- Institutional Review Board, National Hospital Organization Hokkaido Cancer Center, Sapporo, Japan
- Ethics Committee, Asan Medical Center, Seoul, Korea
- Ethics Committee, Samsung Medical Center, Seoul, Korea
- Seoul National University Hospital IRB, Seoul National University Hospital, Seoul, Korea
- IRB of Korea University Guro Hospital, Korea University Guro Hospital, Seoul, Korea
- IRB of Seoul National University Bundang Hospital, Seoul National University Bundang Hospita;, Gyeonggi-do, Korea
- Ethics Committee for Clinical and Other Trials Related to Medicines and Medical Devices, MALMED, Skopje, Macedonia (2 sites)
- Medical Research and Ethics Committee (MREC) Institute for Health Management, Kuala Lumpur, Malasyia (2 sites)
- C. Cientifico De Bioetica E Invest. ISSEMYM Toluca, Toluca, Mexico
- Comité Bioético Para La Investigación Clínica, Centro Oncológico Estatal, Toluca, Mexico
- Comite de Etica en Investigacion de Mexico Centre for Clinical Research, Mexico Centre for Clinical Research S.A. de C.V., Mexico City, Mexico
- Comité de Ética, Investigación y de Bioseguridad, Facultad de Medicina y Hospital Universitario “Dr. José Eleuterio González” de la Universidad Autónoma de Nuevo León, Monterrey, Mexico
- Comite De Etica De Torre Medica Del Pacifico, Torre Medica Del Pacifico, Acapulco, Mexico
- Subcomité de Investigación del Centenario Hospital Miguel Hidalgo, Centenario Hospital Miguel Hidalgo, Aguascalientes, Mexico
- Northern A Health and Disability Ethics Committee, Ministry of Health, Wellington, New Zealand

- Comité Nacional de Bioética, Instituto Conmemorativo Gorgas de Estudios de la Salud, Panama City, Panama
- C. de E. e Invest. del Hospital Regional Cayetano, Hospital Regional Cayetano, Piura, Peru
- Comité Institucional de Etica en Investigación de la Asociación Benéfica Prisma, Asociación Benéfica Prisma, Lima, Peru
- Comité de Etica del Hospital Rebagliati, Hospital Rebagliati, Lima, Peru
- Comité De Ética En Investigación – Arequipa, Hospital Nacional Carlos Alberto Seguín Escobedo, Arequipa, Peru
- Cardinal Santos Medical Center – IRB, Cardinal Santos Medical Center, Manila, Phillipines
- Instituional Ethics and Review Board, Perpetual Succour Hospital, Cebu City, Phillipines
- Komisja Bioetyczna przy Instytucie Centrum Onkologii w Warszawie, Instytucie Centrum Onkologii w Warszawie, Warsaw, Poland (5 sites)
- Comissão de Ética para Investigação Clínica (CEIC), Lisbon, Portugal
- Comisia Nationala de Etica; Prof. Dr. Ion Chiricuta Institute of Oncology, Coltea Hospital, and CLUJ Clinical County Hospital, Bucharest, Romania (3 sites)
- Ethics Committee, Blokhin Cancer Research Center, Moscow, Russia
- Ethics Committee, Ivanovo Regional Clinical Oncology Dispensary, Ivanovo, Russia
- Ethics Committee, State Budget Institution of Healthcare Samara Regional Clinical Oncology Dispensary, Samara, Russia
- Ethics Committee, Tula Regional Clinical Hospital, Tula, Russia
- Ethics Committee, State Budget Institution of Healthcare of Stavropol region Pyatigorsk Oncology Dispensary, Pyatigorsk, Russia
- Ethics Committee, Stavropol Oncology Dispensary, Stavropol, Russia
- Ethics Committee, Moscow City Oncology Hospital #62, Moscow, Russia
- Ethics Committee, SI of Healthcare Kazan Oncology Dispensary, SI of Healthcare Kazan Oncology Dispensary, Kazan, Russia
- Comité de Investigación Clínica, Hospital Generela Universitario Gregorio Maranon, Madrid, Spain (10 sites)
- Regionala etikprövningsnämnden i Lund, University Hospital MAS, Malmo, Sweden
- Ethikkommission Nordwest- und Zentralschweiz (EKNZ), Basel, Switzerland
- Kantonale Ethikkommission Zürich (KEK), Kanton Zürich Gesundheitsdirektion, Zürich, Switzerland (2 sites)

- Research Ethics Committee, National Taiwan University Hospital, Taipei, Taiwan
- Tri-Service General Hospital Institutional Review, Tri-Service General Hospital, Taipei, Taiwan
- IRB, Changhua Christian Hospital, Changhua, Taiwan
- IRB, Kaohsiung Medical University Hospital, Kaohsiung, Taiwan
- Ethics Committee, National Cancer Institute, Bangkok, Thailand
- The Ethics Committee, Rajavithi Hospital, Bangkok, Thailand
- IHRP Ethics Committee, Ministry of Public Health, Nonthaburi, Thailand (2 sites)
- Ethical Clearance Committee on Human Rights, Related to Researches Involving Human Subjects, Faculty of Medicine, Ramathibodi Hospital, Bangkok, Thailand
- Research Ethics Committee, Faculty of Medicine, Chiang Mai University, Chaing Mai, Thailand
- Siriraj Institutional Review Board, Siriraj Hospital, Bangkok, Thailand
- Songklanagarind Ethics Committee, Prince of Songkla University, Songkla, Thailand
- Cukurova Universitesi Tip Fakultesi Etik Kurulu, Adana, Turkey
- NRES Committee East Midlands - Nottingham 2, Health Research Authority, Nottingham, United Kingdom (13 Sites)
- Advarra, Cincinnati, OH, USA
- RCRC Independent Review Board, Austin, TX, USA
- Chesapeake Research Review, Columbia, MD, USA
- Western Institutional review Board, Olympia, WA, USA (28 sites)
- Dartmouth Hitchcock Medical Center IRB, Dartmouth Hitchcock Medical Center, Lebanon, NH, USA
- St. John's Mercy Medical Center IRB, St. John's Mercy Medical Center, Saint Louis, MI, USA
- Kaiser Permanente Northern California IRB, Kaiser Permanente Northern California, Oakland, CA, USA
- Ingalls Memorial Hospital Institutional Review Board, Ingalls Memorial Hospital, Harvey, IL, USA
- Medical University of South Carolina IRB, Medical University of South Carolina, Charleston, SC, USA
- Avera Institutional Review Board, Avera Medical Oncology and Hematology, Sioux Falls, SD, USA
- Institutional Review Board, The University of Texas M.D. Anderson Cancer Center, Houston, TX, USA
- Anne Arundel Medical Center IRB, Anne Arundel Medical Center, Annapolis, MD, USA

- Decatur Memorial Hospital Institutional Review Board, Decatur Memorial Hospital, Decatur, IL, USA
- University of Minnesota IRB, University of Minnesota, Minneapolis, MN, USA
- UCSD Human Research Protections Program, University of California San Diego (UCSD), San Diego, CA, USA
- IUPUI, Indiana University, Indianapolis, IN, USA
- Committee On Human Studies, University of Hawaii, Honolulu, USA
- Mercy IRB, Mercy College, Springfield, MO, USA
- Loyola University Chicago Health Sciences Division (LUCHSD) IRB, Loyola University Chicago, Maywood, IL, USA
- Yale University Human Research Protection Program, Yale University, New Haven, CT, USA
- University of Texas Southwestern, University of Texas Southwestern, Dallas, TX, USA
- University of Chicago Biological Sciences Division IRB, University of Chicago, Chicago, IL, USA
- Vanderbilt University Institutional Review Board, Vanderbilt University, Nashville, TN, USA
- Weill Cornell Medical Center IRB, Weill Cornell Medical Center, New York, NY, USA
- Spectrum Health Research and Human Rights Committee, Spectrum Health, Grand Rapids, MI, USA
- Kaiser Permanente Southern California, Kaiser Permanente Southern California, Pasadena, CA, USA
- Human Subject Research Office, University of Miami, Miller School of Medicine, Miami, FL, USA
- Human Subjects Protection Office, Penn State College of Medicine, Hershey, PA, USA
- Program for Protection of Human Subjects, Mount Sinai School of Medicine, New York, NY, USA
- Sanford Institutional Review Board, Sanford Roger Maris Cancer Center, Fargo, ND, USA (2 sites)
- Mayo Clinic Institutional Review Board, Mayo Clinic, Rochester, MN, USA
- University of Louisville Human Subject Protection, University of Louisville, Louisville, KY, USA
- University of Arkansas for Medical Science IRB, University of Arkansas for Medical Science, Little Rock, AR, USA
- Peoria Institutional Review Board, University of Illinois College of Medicine- Peoria, Chicago, IL, USA
